# High-Dose Ibuprofen in Cystic Fibrosis

**DOI:** 10.3390/ph3072213

**Published:** 2010-07-13

**Authors:** Larry C. Lands, Nurlan Dauletbaev

**Affiliations:** 1 Division of Respiratory Medicine, Montreal Children’s Hospital, D-380, 2300 Tupper Street, Montreal, Quebec, H3H 1P3, Canada; 2 Research Institute of McGill University Health Centre, Montreal Children’s Hospital, C-1223, 2300 Tupper Street, Montreal, Quebec, H3H 1P3, Canada; E-Mail: nurlan.dauletbaev@muhc.mcgill.ca (N.D.)

**Keywords:** ibuprofen, interleukin-8, neutrophils, cystic fibrosis, CFTR

## Abstract

Cystic Fibrosis (CF) is the most common lethal genetic disorder in North America and Europe. Most patients succumb to progressive lung disease characterized by an exaggerated neutrophilic inflammation. In animal models of chronic infection, high-dose ibuprofen was demonstrated to reduce inflammation without hindering bacterial clearance. This led to two clinical trials, which demonstrated a benefit in slowing the progression of lung disease in CF. However, concerns about potential adverse effects have limited the use of high-dose ibuprofen in CF patients. There are a variety of potential mechanisms to account for the observed clinical benefit. A better understanding of these mechanisms could potentially lead to more targeted and better-tolerated anti-inflammatory therapies.

## 1. Introduction

Cystic Fibrosis (CF) is the most common autosomal recessive life-limiting illness in North America and Europe [1]. CF is caused by a mutation in the gene coding for Cystic Fibrosis Transmembrane Regulator (CFTR) protein on chromosome 7. While more than 1,600 mutations in CFTR have been described, close to 70 % of North American and European patients carry the deltaF508 mutation [2]. The median age of survival is progressively increasing, currently being over 40 years of age in many jurisdictions.

Many organs are affected by CFTR mutations; however, pathological changes in the lung are the primary cause of death. Lung function is the single most important prognostic factor. Abnormal CFTR is believed to cause, or contribute to, excessive inflammation in airways of patients with CF. It is not quite clear yet whether the exuberant inflammation predisposes the airways for chronic infections or aggravates the course of these infections. However, it is widely recognized that, in CF, the vicious cycle of infection and inflammation leads to deterioration of lung function and, eventually, respiratory failure. While any bacterial stimulus will cause an exaggerated neutrophilic response [3], chronic infection with *Pseudomonas aeruginosa* is a particularly bad prognostic factor [4,5,6]. The principles of respiratory therapy consist of airway clearance techniques, liquefying respiratory secretions through enzymatic digestions or hydration, and suppression of bacteria. 

With the recognition that airway inflammation plays a significant pathogenic role, modulation of the inflammatory response has become a significant therapeutic target. The archetypical anti-inflammatory therapy, systemic corticosteroids, has been tried [7]. Corticosteroid administration showed positive effects on lung function; however, there were accompanying adverse effects including growth retardation, onset of diabetes and cataract formation, which led to premature stoppage of clinical trials [8].

Studies by Konstan and colleagues suggested high-dose ibuprofen as an alternative to corticosteroids. Their early work using a rodent model of *Pseudomonas aeruginosa* lung infection demonstrated that high-dose ibuprofen could reduce inflammatory changes in the lung without affecting bacterial clearance [9]. This led to clinical trials in patients with CF and the use of high-dose ibuprofen in patients with CF. Clinical use of ibuprofen in CF and potential mechanisms of action will be discussed in the present review. 

## 2. Inflammation and CF Lung Disease

Lung disease in CF is characterized by exaggerated chronic neutrophilic inflammation [10,11,12,13,14] that leads to lung damage, worsening of lung function, and premature death [15,16]. Abnormal CFTR can contribute to, or initiate, this inflammation through several mechanisms. 

First, CFTR mutations may predispose respiratory epithelium to spontaneous overproduction of inflammatory cytokines. The molecular mechanisms of this phenomenon are not fully understood yet, but may involve an increased susceptibility of Nuclear Factor (NF-) κB to activation [17]. Under normal conditions, NF-κB is sequestered in the cytosol bound to its inhibitor, IκB. Cell stimulation results in phosphorylation, and ultimately degradation, of IκB. NF-κB dissociates from phosphorylated IκB and translocates from the cytosol to the nucleus, where it binds to NF-κB binding sites on regulatory sequences of pro-inflammatory genes, such as Interleukin (IL) -8. The binding of NF-κB to its binding site initiates transcription. Other transcription factors, such as C/EBP homologous protein (CHOP), may also be involved in hyperinflammatory response of CFTR-deficient respiratory epithelium [18].

Besides overproduction of inflammatory cytokines immediately after stimulation, CFTR-deficient cells may fail to terminate the inflammatory response [19,20,21]. This could be due to relative deficiency in counter-inflammatory factors, such as Peroxisome Proliferator-Activated Receptors [22,23,24,25] or IL-10 [26,27,28,29].

Further, CFTR is an electrolyte channel, and its malfunction is believed to affect the electrolyte composition of airway surface fluid (ASF). The altered ASF facilitates bacterial growth [30]. Chronic infection establishes itself early on in CF airways [31], and bacterial pathogens stimulate overproduction of inflammatory cytokines including IL-8. 

IL-8 is a major neutrophil chemoattracting agent [32,33] and is present at high levels in CF airways. Respiratory epithelial cells are the primary source of IL-8 in the lungs, although other cell types, including macrophages and neutrophils, can contribute [33]. Local production of IL-8 establishes a chemotactic gradient leading to recruitment of neutrophils from the bloodstream [34,35]. The importance of epithelial IL-8 production in CF is highlighted by two distinct animal models: 1) naïve sterile human lung grafts in severe combined immunodeficiency mice secrete IL-8 and recruit neutrophils to the CF lungs causing tissue damage [36]; 2) delta F508 cystic fibrosis mice raised in a pathogen-free environment spontaneously have increased levels of lung lavage fluid neutrophils and MIP-2, a mouse analog to human IL-8 [37]. 

Recruited neutrophils produce proteases (e.g., neutrophil elastase) and oxidants that damage respiratory epithelium and sustain hyperactivation of NF-κB and production of IL-8. Extracellular elastase also induces mucin overproduction by epithelial cells and cleaves phagocytic receptors on macrophages [38]. This protease activity goes largely unimpeded as released oxidants damage antiproteases, leading to an excess of free protease activity and decreased killing of *Pseudomonas aeruginosa* by neutrophils [38,39,40,41,42,43,44,45]. 

While NF-κB is the principal activator of IL-8 production, other transcription factors, such as Activating Protein (AP)-1 [46] and Mitogen-Activated Protein Kinases (JNK, p38 MAPK, and ERK) [47] are involved in up-regulation of IL-8 in response to CF-relevant stimuli. These stimuli include pro-inflammatory cytokines [e.g., Tumour Necrosis Factor (TNF) -α and IL-1β], microbial products (e.g., LPS, *Pseudomonas aeruginosa* pilin, flagellin, pyocyanin), proteases, and products resulting from oxidative stress [e.g., 4-hydroxy-2-nonenal (4-HNE), a lipid peroxidation product]. The inflammatory stimuli up-regulate IL-8 production through a multitude of signaling pathways [48,49,50]. 

JNK, ERK, and p38 lead to AP-1 activation via phosphorylation [47]. JNK also plays a role in NF-κB transactivation [47]. p38 can also enhance recruitment of NF-κB to its binding site on the IL-8 promoter through phosphorylation of histone H3 [51]. Phosphorylation of the histones leads to histone acetylation and results in uncoiling of the chromatin, making it available for transcription. p38 also regulates the activity of CBP/p300, which acts to stabilize and link transcription factors and coactivators with the transcriptional machinery, partially through innate acetylation activity [52]. Moreover, p38 enhances IL-8 production by stabilizing IL-8 mRNA [47,53].

## 3. Clinical Use of Ibuprofen in Cystic Fibrosis

To date, there have been two large-scale trials of high dose ibuprofen in CF patients [54,55]. The first clinical trial was reported by Konstan and colleagues in 1995 [56]. Over a 4-year period, 84 children over the age of 5 and adults who had mild lung disease [Forced Expiratory Volume in 1-second (FEV_1_)>60% predicted] were randomized to receive either high-dose ibuprofen or placebo.

The dose (generally 20–30 mg/kg) was adjusted to reach a peak plasma concentration of 50–100 μg/mL, and this was administered twice daily. Peak plasma concentrations are reached within three hours of oral dosing [57]. Konstan’s group, using a mouthwash method to assess neutrophil recruitment, demonstrated that plasma concentrations below 50 μg/mL actually increased neutrophil influx to mucosal surfaces. Specifically, levels below 50 μg/mL resulted in higher neutrophil counts in the mouthwashes [58]. A concentration range of 50–100 μg/mL is required to inhibit neutrophil migration [58]. Plasma concentrations above 100 μg/mL are associated with increased adverse events. 

For the entire group, there was a significant and important decrease in the annual rate of decline in lung function (FEV_1_ % predicted). In a *post-hoc* analysis, it was found that the effect was seen in those patients under the age of 13 years at the beginning of the trial (an 89% reduction in the annual rate of decline in FEV_1_ % predicted), while no significant effect was seen in adult patients. There were additional beneficial effects, including maintenance of body weight, another important prognostic factor, fewer hospitalizations, and improved chest radiograph scores. There were only limited adverse effects seen, although one subject withdrew due to epistaxis and one for conjunctivitis. There were surprisingly few gastrointestinal complications.

A second two-year trial was conducted in Canada focusing on patients 6–18 years of age, with FEV_1_ > 60% predicted [59]. In this study of 142 patients, there was a non-significant 45% decrease in the annual rate of decline in FEV1 % predicted. However, there was a significant decrease in the annual rate of decline of Forced Vital Capacity (FVC % predicted). More patients withdrew in the placebo group than treatment group. However, there was one case of tinnitus and one major gastrointestinal bleed. Partway through the trial, due to changes in practice, routine gastrointestinal protection with a H2-blocker or protein ion pump inhibitor was recommended. Currently, protein ion pump inhibitors are recommended as a concurrent therapy.

It needs to be noted that in the Canadian study, it was recommended that study drug be discontinued during any time period that patients would receive intravenous aminoglycosides. There are several reports of acute severe nephrotoxicity when high-dose ibuprofen is continued during administration of intravenous aminoglycosides [60,61].

The data from these two trials were pooled in a meta-analysis [55]. The data supported a beneficial effect on the annual rate of decline of FEV_1_ % predicted in children with mild lung disease.

In order to assess the effect of ibuprofen in the clinical setting without the oversight of a clinical trial, an analysis of patients in the US CF Foundation patient data registry was conducted [62]. Children age 6–17 years with an FEV_1_ > 60% predicted were included. The data also supported that high-dose ibuprofen slowed the rate of progression of lung disease.

Despite the described clinical benefits, ibuprofen is used by relatively small number of patients with CF. The biggest barrier to a frequent use of this drug seems to be a concern over potential adverse effects. Some patients with CF may be more predisposed to adverse effects. There has been a case report indicating that the risk of gastrointestinal adverse effect may be higher in patients with anatomical abnormalities in the gastrointestinal tract or decreased esophageal motility [63]. The incidence of gastrointestinal bleedings is relatively small. A single-center survey of clinical experience with high-dose ibuprofen reported abdominal pain as the most frequently reported adverse effect; gastrointestinal bleeding occurred in a small proportion of patients [64]

The aforementioned analysis of the US CF Foundation patient data registry also addressed the incidence of adverse effects in the ibuprofen-treated patients with CF. The treatment was associated with an increased risk of gastrointestinal bleeding requiring hospitalization, but the number of occurrences was relatively small (annual incidence 0.37 in ibuprofen-treated patients *versus* 0.14 in untreated patients) [62]. It was concluded that the clinical benefits appear to outweigh the risks in the therapy with high-dose ibuprofen [54].

Presently, CF patients undergo a 3-hour pharmacokinetic study prior to starting high-dose ibuprofen. This should be done using the brand and dosage strength that the patient intends on using, as this may influence the results of the study. Typically, studies are conducted in the morning. A standard dose of 20–30 mg/kg (maximum 1,600 mg) is administered at least two hours after eating, and hourly blood samples are drawn. The aim is to have a peak plasma concentration of 50–100 μg/mL on at least one of the three hourly measurements. The concentrations are typically measured by high-pressure liquid chromatography. There are limited facilities with this capability, but a US national service does exist (Case Western Reserve University School of Medicine, Division of Clinical Pharmacology, E-Mail: cfibuplab@po.cwru.edu; telephone: +1 216 844-8433).

Pharmacokinetics are repeated every two years, or sooner if a 25% weight change occurs. For safety monitoring, the annual blood testing and urine analysis that are already recommended for routine care in CF is generally enough. Recommendations have been made to check for occult blood in the stool every three months, but this is likely to give rise to many false positive results [54].

To conclude, high-dose ibuprofen is an effective medication slowing down a decline in lung function, with the highest efficacy in younger patients with mild CF lung disease. The clinical benefits appear to outweigh the risk of adverse effects.

## 4. Ibuprofen and Cystic Fibrosis Lung Disease

As discussed, studies in animal models of chronic infection suggested that high-dose ibuprofen could reduce the inflammatory response without impairing bacterial clearance. The clinical studies supported that this translates into a clinical benefit. However, concerns about potential adverse effects have limited the use of high-dose ibuprofen [65]. A better understanding of the mechanisms of action of high-dose ibuprofen could then lead to potent, but safer, anti-inflammatory agents for CF.

High-dose ibuprofen reduced recruitment of neutrophils in both healthy individuals and CF patients, provided the peak plasma concentration was above 50 μg/mL [58]. It is believed that the effect of ibuprofen at these concentrations is beyond suppression of prostaglandin synthesis [66]. For instance, in the rodent *Pseudomonas aeruginosa* model, the suppression of neutrophil recruitment was associated with reduced concentrations of Leukotriene (LT)-B4, a neutrophil-derived chemoattractant [9]. Surprisingly, a study validating induced sputum as an outcome measure for clinical trials could only demonstrate a trend to reduced percentage of sputum neutrophils after 1-month of high-dose ibuprofen therapy [67]. This raises the question of where high-dose ibuprofen is exerting its positive clinical effects.

Anti-inflammatory effects of ibuprofen have traditionally been attributed to inhibition of cyclooxygenases which control synthesis of prostaglandins. Since prostaglandins are mediators of inflammation, suppression of prostaglandin synthesis was believed to be the principal anti-inflammatory mechanism of ibuprofen and other nonsteroidal anti-inflammatory drugs. Only recently, it was observed that ibuprofen at high doses (low to mid millimolar range) can suppress transcriptional activity of NF-κB and other pro-inflammatory transcription factors [66]. In CF, ibuprofen is administered to achieve low millimolar concentrations in blood plasma. These concentrations could theoretically exert anti-inflammatory effects in CF by suppressing NF-κB and, thus, NF-κB dependent inflammatory genes, such as IL-8. In an investigation of potential mechanisms of ibuprofen in CF, a study to explore the effects of ibuprofen on respiratory epithelial production of IL-8 was conducted [68]. In this study, an ibuprofen concentration in the plasma therapeutic range (100 μg/mL or 0.48 mM) was utilized. In CFTE29o-, a patient-derived immortalized tracheal epithelial cell line expressing deltaF508 CFTR, both TNF-α and IL-1β stimulated secretion of IL-8. Ibuprofen suppressed stimulated NF-κB transcriptional activity, as measured by a luciferase gene reporter assay, but did not decrease stimulated IL-8 mRNA expression or IL-8 secretion. This failure to suppress IL-8 expression is compatible with the results for induced sputum [67]. It was concluded that the observed suppression of NF-κB transcriptional activity by high-dose ibuprofen observed in this and other studies [69,70] was not sufficient to reduce IL-8 expression. Thus, alternative mechanisms must explain the positive clinical effects of high-dose ibuprofen in CF. 

Potential alternative mechanisms of action in CF include inactivation of C/EPB homologous protein (CHOP) in CF respiratory epithelial cells [18]. CHOP is a transcription factor that results in chronic overproduction of IL-8 production in the absence of stimulation. CHOP is induced by stress in the endoplasmatic reticulum (ER). In CF, the deltaF508 mutation results in misfolding of the CFTR, leading to accumulation in the ER. This results in ER engorgement and stress. CHOP is also induced by prostaglandin E_2_ (PGE_2_). The PGE_2_ pathway can be suppressed by nonsteroidal anti-inflammatory agents [18]. While high-dose ibuprofen did not appear to suppress IL-8 secretion in respiratory epithelial cells stimulated with TNF-α or IL-1β, it is possible that high-dose ibuprofen could exert anti-IL-8 effects during stable periods in CF patients. High-dose ibuprofen could also potentially decrease inflammation through down-regulation cAMP activation in CF respiratory epithelial cells [18]. 

High-dose ibuprofen can also enhance the function of delta F508 CFTR [71]. Rectification of CFTR function can lead to a decreased inflammation [72]. Alternatively, high-dose ibuprofen may directly target neutrophils and neutrophil activities, such as migration [73].

Stimulation of Peroxisome Proliferator-Activated Receptors (PPARs) by ibuprofen could potentially be another molecular mechanism of its anti-inflammatory effects in cystic fibrosis. The PPARs are members of the nuclear-hormone-receptor superfamily [74]. They bind a variety of ligands and modify cell responses by activating or suppressing expression of PPAR-sensitive genes. One of the PPAR isoforms, PPAR-γ, is a negative regulator of inflammatory responses; its expression is down-regulated in CFTR-deficient tissues [24]. Further, CFTR-deficient cells express high levels of tissue transglutaminase (TG2) that alters the subcellular localization of PPAR-γ and thus blocks its anti-inflammatory effects [25]. The expression of PPAR-γ can be stimulated by ibuprofen [75]. Importantly, stimulation of PPAR-γ expression requires concentrations of ibuprofen higher than those needed to inhibit COX [76]. The high ibuprofen concentrations used to treat CF lung disease may be sufficient to stimulate PPAR-γ expression. 

## 5. Conclusions

CF lung disease is characterized by a dysregulated neutrophilic inflammation. High-dose ibuprofen has been demonstrated to clinically slow the progression of lung disease in CF, but concerns about potential adverse events have limited its use in CF patients. There are a variety of potential mechanisms responsible for this clinical effect. A direct suppression of respiratory epithelial secretion of IL-8, the most potent chemoattractant for neutrophils to the lungs, does not appear to be the most likely mechanism. A better understanding of the mechanisms responsible for the clinical benefits of existing drugs is needed to develop newer, more targeted and safer anti-inflammatory therapies. 
